# Effect of respiratory pattern on automated clinical blood pressure measurement: an observational study with normotensive subjects

**DOI:** 10.1186/s40885-017-0071-3

**Published:** 2017-07-18

**Authors:** Natalia Herakova, Nnenna Harmony Nzeribe Nwobodo, Ying Wang, Fei Chen, Dingchang Zheng

**Affiliations:** 1 Health and Wellbeing Academy, Faculty of Medical Science, Anglia Ruskin University, Chelmsford, UK; 2 Department of Computer Engineering, faculty of Engineering, Enugu State University of Science and Technology, Enugu, Nigeria; 3 Department of Electrical and Electronic Engineering, Southern University of Science and Technology, Shenzhen, China

**Keywords:** Blood pressure, Breathing pattern, Diastolic, Exhalation, Fast-deep breathing, Inhalation, Respiratory pattern, Slow-deep breathing, Systolic

## Abstract

**Background:**

It has been reported that deep breathing could reduce blood pressures (BP) in general. It is also known that BP is decreased during inhalation and increased during exhalation. Therefore, the measured BPs could be potentially different during deep breathing with different lengths of inhalation and exhalation. This study aimed to quantitatively investigate the effect of different respiratory patterns on BPs.

**Methods:**

Forty healthy subjects (20 males and 20 females, aged from 18 to 60 years) were recruited. Systolic and diastolic BPs (SBP and DBP) were measured using a clinically validated automated BP device. There were two repeated measurement sessions for each subject. Within each session, eight BP measurements were performed, including 4 measurements during deep breathing with different respiratory patterns (Pattern 1: 4.5 s vs 4.5 s; Patter 2: 6 s vs 2 s; Pattern 3: 2 s vs 6 s; and Pattern 4: 1.5 s vs 1.5 s, respectively for the durations of inhalation and exhalation) and additional 4 measurements from 1 min after the four different respiratory patterns. At the beginning and end of the two repeated measurement sessions, there were two baseline BP measurements under resting condition.

**Results:**

The key experimental results showed that overall automated SBP significantly decreased by 3.7 ± 5.7 mmHg, 3.9 ± 5.2 mmHg, 1.7 ± 5.9 mmHg and 3.3 ± 5.3 mmHg during deep breathing, respectively for Patterns 1, 2, 3 and 4 (all *p* < 0.001 except *p* < 0.05 for Pattern 3). Similarly, the automated DBPs during deep breathing in pattern 1, 2 and 4 decreased by 3.7 ± 5.0 mmHg, 3.7 ± 4.9 mmHg and 4.6 ± 3.9 mmHg respectively (all *p* < 0.001, except in Pattern 3 with a decrease of 1.0 ± 4.3 mmHg, *p* = 0.14). Correspondingly, after deep breathing, automated BPs recovered back to normal with no significant difference in comparison with baseline BP (all *p* > 0.05, except for SBP in Pattern 4).

**Conclusions:**

In summary, this study has quantitatively demonstrated that the measured automated BPs decreased by different amounts with all the four deep breathing patterns, which recovered back quickly after these single short-term interventions, providing evidence of short-term BP decrease with deep breathing and that BP measurements should be performed under normal breathing condition.

## Background

The importance of accurate blood pressure (BP) measurement is without doubt. According to a major review in the Journal of the American Medical Association (JAMA), a 5 mmHg error would result in 21 million Americans being denied treatment or 27 million being exposed to unnecessary treatment, depending on the direction of the error [1]. Unfortunately, BP measurement is still one of the most poorly performed diagnostic measurements in real clinical practice [2]. It is generally accepted that BP measurement inaccuracies are associated with the measurement conditions, including incorrect patient posture, incorrect arm position and incorrect cuff position and size [3–5], and also associated with short-term physiological changes during the measurement leading to within-subject BP variability [6]. It has been widely accepted that respiration is one of the key factors affecting short-term physiological changes in BP and therefore leading to potential measurement error [2].

Respiration is the natural physiological mechanism during which air is inhaled into the lungs and then exhaled via the nose or mouth. Normal breathing is involuntary and rhythmic, and two processes are involved during breathing are inspiration (or inhalation) and expiration (or exhalation) [7, 8]. During inspiration with oxygen inhaled into the body, the intercostal muscles contract, expanding the ribcage and the diaphragm contracts, pulling down to increase the volume of the chest. This lowers the pressure inside the thorax and gets air sucked into the lungs. During exhalation with carbon dioxide exhaled out of the body, the intercostal muscles relax and lower the ribs downward, causing the diaphragm to relax and move back upwards. This causes a decrease in thorax volume, which as a result, increases the pressure inside the thorax.

Several published studies have shown that respiration influences both short-term and long-term systolic and diastolic blood pressures (SBP and DBP) measured by different techniques [6, 9–14]. For instance, it has been reported by Zheng et al. [6] that, with regular slow and deep breathing, both manual auscultatory SBP and DBP decreased significantly by 4.4 and 4.8 mmHg respectively, in comparison with normal breathing. On the other hand, the physiological mechanisms of respiration process indicate that BP is decreased during inhalation and increased during exhalation [15]. Since a single BP measurement may take more than one or two normal respiratory cycles, the measured BPs could be potentially different with different types of deep breathings where various lengths of inhalation and exhalation are involved. To the best of our knowledge, there is little quantitative information available on the effect of different respiratory patterns on measured BPs.

The aim of this research was to quantitatively investigate the effect of different breathing patterns on BPs in comparison with baseline BP measurement.

## Methods

### Subjects

Forty healthy normotensive subjects, 20 males and 20 females, aged 18–60, were recruited. The requirements of inclusion criteria included: normal healthy individual, age range 18–60 years old, with SBP < 140 mmHg and DBP < 90 mmHg. Participants with known hypertension and antihypertensive medical treatment, or cardiovascular disease, such as ischaemic heart disease, congestive heart failure, chronic atrial fibrillation, renal failure and previous stroke, were excluded. Additionally, if the initial BP measurement showed SBP > 140 mmHg and DBP > 90 mmHg, these participants were also excluded. Subjects’ demographic information, including age, weight, height and arm circumference are summarized in Table 1.

**Table 1:** Demographic data of subjects studied

Subject information	Minimum	Maximum	Mean	Standard deviation
Age	20	59	37	10
Height (cm)	151	183	170	9
Weight (kg)	51	108	74	14
Arm circumference (cm)	24	40	31	4

This study has been reviewed and approved by the Faculty Research Ethics Panel, Faculty of Medical Science, Anglia Ruskin University. The investigation conformed with the Declaration of Helsinki, and all subjects gave their written informed consent to participate in the study.

### Blood pressure measurement protocol and procedure

The measurements were conducted in a quiet room at Anglia Ruskin University. All the subjects were asked to rest in a seated position for at least 5 min before the formal BP measurement. SBP, DBP were measured from the left arm using a suitable cuff matched to individual arm circumference (adult 24–34 cm; large adult 34–41 cm) by a clinically validated automated BP device (HBPM Omron, M6 Comfort). The HR value was also obtained during each measurement from the device. The BP measurement procedure followed the Measurement Guideline from the European Society of Hypertension [16].

**Fig. 1:**
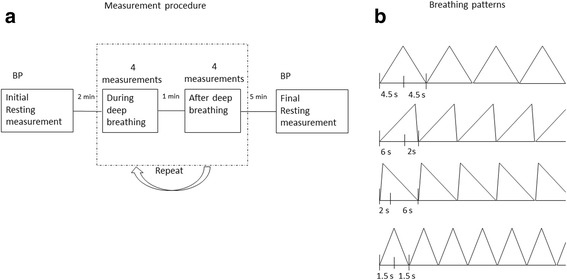
**a** BP measurement procedure. Participants were given 5 min before the initial BP was measured. During deep breathing, before the automated BP measurements started, 3 respiratory cycles were performed, and this continued until the completion of BP measurement. **b** Illustration of deep breathing with four different respiratory patterns. Pattern 1: slow breathing (↑4.5 s ↓4.5 s); Pattern 2: long inspiration followed by short expiration (↑6 s ↓2 s); Pattern 3: short inspiration followed by long expiration (↑2 s ↓ 6 s); Pattern 4: fast breathing (↑1.5 s ↓1.5 s)

A mobile phone application (Paced Breathing, Android App on Google Play), which was designed to adjust the duration of inhalation and exhalation and display the visual pattern on the screen (see Fig. 1b), was used for the subjects to follow the different respiratory patterns and synchronize their breathing with the defined patterns. All subjects were given the opportunity to practice and be familiar with these respiratory patterns before the formal experiment.

There were two repeated measurement sessions for each subject (see Fig. 1a). At the beginning and end of the two sessions, there were two baseline BP and HR readings under resting condition. Within each session, eight BP and HR measurements were performed, including 4 measurements during deep breathing using four different respiratory patterns and additional 4 measurements from 1 min after the four different patterns of deep breathing. During deep breathing with a certain respiratory pattern, before the automated BP measurement started, the subjects were asked to breathe three respiratory cycles, and this continued until the BP measurement completed. The order of the sequence of the four respiratory patterns was randomised between subjects. As shown in Fig. 1b), the details of the four respiratory patterns were:

- Pattern 1: slow deep breathing with 4.5 s of inhalation and 4.5 s exhalation;
- Pattern 2: long inspiration followed by short expiration with 6 s inhalation and 2 s exhalation;
- Pattern 3: short inspiration followed by long expiration with 2 s inhalation and 6 s exhalation;
- Pattern 4: fast deep breathing with 1.5 s inhalation and 1.5 s exhalation.

### Data and statistical analysis

All recorded BP and HR data were stored in Excel Spreadsheet, then transferred and analysed in statistical software SPSS 20.0. The means and standard deviations (SDs) of SBP, DBP and HR were calculated separately for the baseline, during four different respiratory patterns, and after deep breathings. Analysis of variance (ANOVA) was then performed to investigate the measurement repeatability and the effect of respiratory pattern on BP and HR measured during and after deep breathing. The post-hoc multiple comparison in the ANOVA test was used to compare the differences in BP and HR between respiratory patterns. A *p* value below 0.05 was considered statistically significant.

## Results

### BP and HR measurement repeatability

ANOVA analysis showed that baseline BP and HR at the beginning and end of the main measurement sessions were repeatable (*p* = 0.4 for SBP, *p* = 0.5 for DBP and *p* = 0.6 for HR). Furthermore, BP and HR measurements during and after deep breathing were also repeatable between the repeat sessions (all *p* > 0.1). As the BP and HR measurements were repeatable, their average values from the two repeat measurements were used as a reference value for each subject.

### HR changes during and after deep breathing in comparison with baseline

**Fig. 2:**
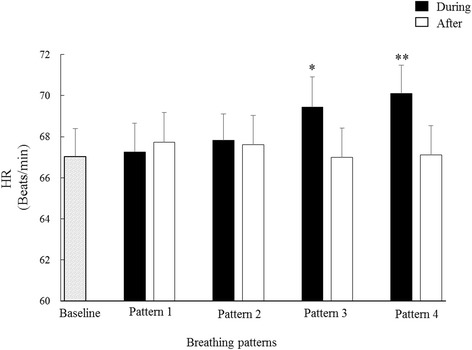
Means + SDs of HR measured during and after deep breathing, separately for different respiratory patterns. ^**^ *p* <0.001; ^*^ *p* <0.05 in comparison with baseline HR

Figure 2 shows the HRs measured during and after deep breathing. In comparison with the Baseline, it can be seen that HR during deep breathing increased significantly in Patterns 3 and 4 (69.4 ± 9.3 and 70.1 ± 8.7 vs 67.1 ± 8.5 beats/min, both *p* < 0.01), but not in Patterns 1 and 2. After deep breathing, all the HRs recovered back to normal with no statistically significant HR difference in comparison with Baseline (all *p* > 0.2).

### SBP changes during and after deep breathing in comparison with baseline

**Fig. 3:**
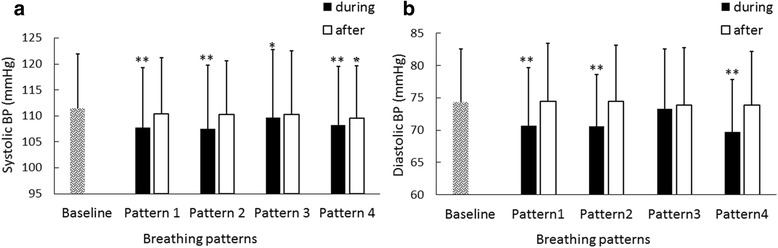
Means + SDs of systolic (**a**) and diastolic (**b**) blood pressures measured during and after deep breathing, separately for different respiratory patterns. ^**^ *p* < 0.001; ^*^ *p* <0.05 in comparison with baseline BP

**Fig. 4:**
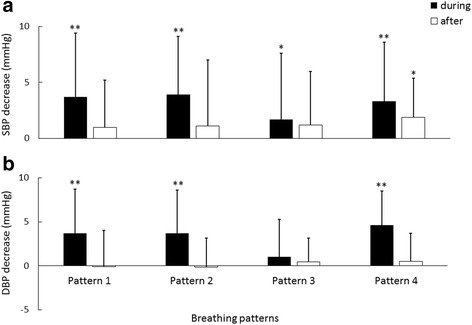
Decrease of systolic (**a**) and diastolic (**b**) blood pressures (SBP and DBP) during and after deep breathing in comparison with baseline. The results for different respiratory patterns are given separately. ^**^ *p* < 0.001; ^*^ *p* < 0.05 in comparison with baseline BP

Figure 3a) shows SBP measured during and after deep breathing. It can be seen that SBPs measured during deep breathing were significantly decreased in comparison with the Baseline. Specifically, as shown in Fig. 4a) and Table 2, SBP in Patterns 1, 2 and 4 decreased by 3.7 ± 5.7 mmHg, 3.9 ± 5.2 mmHg and 3.3 ± 5.3 mmHg respectively (all *p* < 0.001) and SBP in Pattern 3 decreased by 1.7 ± 5.9 mmHg (*p* < 0.05). It was also observed that SBP after deep breathing did not change significantly in comparison with Baseline in Pattern 1, 2, 3 (decreased by 1.0 ± 4.2 mmHg, 1.1 ± 3.5 mmHg, 1.2 ± 4.8 mmHg, with *p* > 0.05).

**Table 2:** Means ± SDs of systolic and diastolic blood pressures (SBP and DBP) measured during and after deep breathing and their differences in comparison with baseline BPs

		During breathing	BP decrease	After breathing	BP decrease
SBP (mmHg)	Baseline	111.5 ± 10.5			
	Pattern 1	107.8 ± 11.5	3.7 ± 5.7 ^**^	110.4 ± 10.7	1.0 ± 4.2
	Pattern 2	107.6 ± 12.2	3.9 ± 5.2 ^**^	110.3 ± 10.3	1.1 ± 3.5
	Pattern 3	109.7 ± 13.0	1.7 ± 5.9 ^*^	110.3 ± 12.2	1.2 ± 4.8
	Pattern 4	108.2 ± 11.3	3.3 ± 5.3 ^**^	109.5 ± 10.2	1.9 ± 3.5^*^
DBP (mmHg)	Baseline	74.4 ± 8.2			
	Pattern 1	70.7 ± 9.0	3.7 ± 5.0 ^**^	74.4 ± 9.0	−0.1 ± 4.1
	Pattern 2	70.7 ± 8.0	3.7 ± 4.9 ^**^	74.5 ± 8.6	−0.1 ± 3.3
	Pattern 3	73.3 ± 9.2	1.0 ± 4.3	73.9 ± 8.8	0.5 ± 2.7
	Pattern 4	69.7 ± 8.2	4.6 ± 3.9 ^**^	73.9 ± 8.3	0.5 ± 3.2

^**^ *p* < 0.001; ^*^ *p* < 0.05

### DBP changes during and after deep breathing in comparison with baseline

Figure 3b) shows DBP measured during and after deep breathing. In comparison with the Baseline, DBP in Patterns 1, 2 and 4 during deep breathing decreased significantly by 3.7 ± 5.0 mmHg, 3.7 ± 4.9 mmHg and 4.6 ± 3.9 mmHg respectively (all *p* < 0.001). DBP in Pattern 3 did not decrease significantly in comparison with Baseline (mean difference of 1.0 ± 4.3 mmHg; *p* = 0.14). DBP after deep breathing did not show significant changes in comparison with Baseline (with the mean decreased of −0.09 ± 4.15 mmHg, −0.14 ± 2.71 mmHg, 0.45 ± 2.71 mmHg and 0.46 ± 3.16 mmHg, respectively for Patterns 1, 2, 3 and 4; all *p* > 0.05).

## Discussion and conclusions

This study quantitatively demonstrated the effect of different deep breathing patterns (with different durations of inhalation and exhalation) on automated BPs. To the best of our knowledge, this was the first study to comprehensively compare the short-term effect of different breathing patterns on automated BPs.

According to the results of the present study, Pattern 1 (slow and deep breathing with 4.5 s inhalation and 4.5 s exhalation) achieved a significant decrease in both automated SBP and DBP by 3.7 ± 5.7/3.7 ± 5.0 mmHg, respectively. These results agreed with the findings from previous studies, where it has been concluded that slow and deep breathing could reduce BP [6, 10, 17, 18]. Some of the published studies mainly focused on the long-term effect, while the others on the short-term effect on BP variability. Bhavanani, et al. [17] applied slow and deep breathing with equal duration of inhalation and exhalation at the rate of 6 breaths/min and achieved BP reduction in hypertensive patients with 5 min of practice. With slow deep breathing, BPs are reduced via increased baroreflex sensitivity, which regulates BP by controlling heart rate, sympathetic activity and chemoreflex activation [19]. In addition, any slight deviation in the oxygen content in the brain may affect the cardiovascular function. During slow and deep breathing, oxygenation allows the body to absorb its full oxygen quota, which relaxes the brain and calms the cardiovascular system, resulting in reduced stress and decreased BP.

This study also showed significant decrease in automated SBP/DBP (3.9 ± 5.2 mmHg/3.7 ± 4.9 mmHg, respectively) in Pattern 2 with 6 s inhalation and 2 s exhalation. This could be explained by the temporary physiological effect of decreasing stroke volume during long standing inhalation or widened thoracic for downward movement of diaphragm [20]. It is also noticed that there was no significant HR change with this breathing pattern, indicating the potential involvement of sympathetic de-activation and that the BP lowering effect could be persistent during deep exhalation.

The respiratory Pattern 3 involved 2 s inhalation and 6 s exhalation. Although some published studies used similar pattern and achieved a positive BP reduction [10, 19, 21], the results of the present study showed that this pattern only achieved significant automated SBP decrease (1.7 ± 5.9 mmHg), but for DBP (1.0 ± 4.3 mmHg). This could be explained by the physiology of long exhalation which relates to a relaxation of diaphragm and an increase of intrathoracic pressure, refilling the left ventricle with blood and causing BP to increase. Pattern 4 was the only patter where the fast breathing (1.5 s inhalation and 1.5 s exhalation) was used. The HR was significantly increased with this pattern. Although many participants complained of light dizziness during the experiment, significant decrease was still observed in SBP/DBP (3.3 ± 5.3/4.6 ± 3.9 mmHg, respectively).

Overall, the four respiratory patterns applied in this study all reduced the short-term BPs by different amounts. It is noticed that the participants felt more comfortable to follow some patterns than the others, indicating different physiological mechanisms could be involved in these patterns. There is also possibility of this BP lowering effect might be from “self-notice” of own respiration or concentration on own respiration regardless of respiratory pattern. Published report has showed transcendental meditation is associated with reductions of SBP and DBP [22]. Similar BP reduction was observed in both normotensive and hypertensive individuals. For a better understanding of their underlying mechanisms, more data about BP reduction efficacy of self-notice or concentration on spontaneous respiration is required in a future study.

In addition, this study also showed that the measured BPs recovered back to normal 1 min after the deep breathings, with no significant difference in comparison with Baseline. The only exception was SBP in Pattern 4 (with mean difference of BP by 1.9 ± 3.5 mmHg), which can be explained by the fact that it takes longer for the cardiovascular circulation to be stabilised. The results provided evidence that, although short-term BP variability was produced during deep breathing, both BP and HR could recover quickly back to normal, suggesting that it is better to measure BP under resting condition with normal breathing pattern to achieve reliable BP values.

One of the limitations in this study is the fact that it was not established whether the participants should inhale/exhale with mouth or nose [17, 20, 23]. A comparison of the effect of using different breathing approaches on BPs could be included in a future study. Next, only the short-term effect of deep breathing on BPs was investigated. The long-term benefit of each breathing pattern with regular practice should be investigated, to confirm whether routinely performed sessions of breathing exercises may lead to a sustained reduction in BP for exploring its potential clinical application. Furthermore, this preliminary study was conducted only on normotensive subjects. The neurohumoral balance could be de-ranged in patients with hypertension. Therefore, it is not guaranteed whether similar results of this research could be achieved with hypertensive patients or patients with other diseases.

It should be also noted that the measured BPs in this study were from a clinically validated automated BP device. The automated BP device used here is based on oscillometric technique, where BPs are normally estimated from the global envelope of the oscillometric pulses recorded from the whole period of the measurement that covers several respiratory cycles. Since the measuring principle of manual auscultatory technique is different with the oscillometric technique, the effect of different respiratory patterns on manual auscultatory BPs could be different, depending on which phase (inspiration or expiration) the SBP and DBP determinations are made [8]. The potential different effects of deep breathing on manual and automated BPs are worth further investigation. It would be also useful to investigate the beat-to-beat BP changes in association with deep breathing with different respiratory patterns.

In summary, this study has quantitatively demonstrated that the measured automated BPs decreased by different amounts with all the four deep breathing patterns, which recovered back quickly after these single short-term interventions, providing scientific evidence of short-term BP decrease with deep breathing and that BP measurements should be performed under normal breathing condition.

## Abbreviations

BP: Blood pressure
DBP: Diastolic blood pressure
SBP: Systolic blood pressure
SD: Standard deviation
